# Sociodemographic moderators of the association between functional limitations and depressive symptoms among Chinese older adults

**DOI:** 10.3389/fpsyt.2025.1509485

**Published:** 2025-08-13

**Authors:** Haijun Hao, Junyue Yue, Yeong Hun Yeo

**Affiliations:** ^1^School of Public Administration (School of Philanthropy), Shandong Technology and Business University, Yantai, China; ^2^Department of Social Welfare, Jeonbuk National University, Jeonju, Republic of Korea

**Keywords:** functional limitations, depressive symptoms, age group, living arrangement, region, Chinese older adults

## Abstract

**Introduction:**

Depressive symptoms among older adults are a growing public health concern, particularly in rapidly aging populations like China. Functional limitations, commonly experienced with advancing age, have been shown to exacerbate depressive symptoms.

**Methods:**

This study investigates the moderating effects of sociodemographic factors (including individual, familial, and society) on the association between functional limitations and depressive symptoms among Chinese older adults, using data from the 2018 Chinese Longitudinal Healthy Longevity Survey (CLHLS). The sample included 9,354 adults aged 65 and above.

**Results:**

Results indicate that age group, living arrangement, and region significantly moderate the relationship between functional limitations and depressive symptoms. Specifically, functional limitations have a greater impact on depressive symptoms among the "young-old" compared to the "oldest-old," are more pronounced among those living alone versus with family, and affect rural residents more than urban ones.

**Discussion:**

These findings underscore the need for targeted interventions that address these moderating factors to improve mental health outcomes among older Chinese adults.

## 1 Introduction

Depressive symptoms are a pervasive mental health concern that disproportionately affects the older adult population, significantly diminishing their overall well-being and quality of life (1, 2). This heightened vulnerability to depressive symptoms in older adults is frequently associated with the increasing prevalence of functional limitations within this demographic (3). Functional limitations refer to difficulties in performing everyday activities due to physical, cognitive, or sensory impairments, which tend to accumulate with age (4). These limitations often encompass both basic activities of daily living (ADLs)—such as eating, dressing, and bathing—and more complex instrumental activities of daily living (IADLs), including tasks like managing finances, preparing meals, or shopping (5). As these limitations worsen, they not only impede an individual’s ability to live independently but also contribute to a heightened risk of mental health issues, particularly depressive symptoms (6). Given the significant impact that depressive symptoms have on both mental and physical health, understanding how functional limitations contribute to depressive symptoms is vital for informing interventions and public health policies aimed at improving the lives of older adults.

A large body of research consistently demonstrates a robust association between functional limitations and depressive symptoms in older adults, underlining the substantial influence of physical health on mental well-being in later life (4, 7, 8). Functional limitations often stem from chronic illnesses or age-related conditions, including arthritis, cardiovascular disease, sensory impairments, and neurological disorders, which reduce the ability of older individuals to engage independently in routine activities, fostering a sense of dependency and loss of autonomy (9). The psychological burden associated with losing independence frequently leads to a decline in self-esteem, feelings of helplessness, and increased anxiety, ultimately resulting in depressive symptoms (10). This loss of autonomy significantly heightens emotional distress among older adults, as it often leads to increased social isolation and reduced participation in community activities, exacerbating depressive symptoms (11, 12). Functional impairments further contribute to emotional and psychological burdens for older adults, particularly as these limitations become cumulative over time and lead to increased reliance on others for daily activities (13). As these challenges accumulate, they underscore the importance of addressing social and mental health interventions tailored to mitigate the adverse effects of functional decline on emotional well-being in older populations.

The relationship between functional limitations and depressive symptoms, however, is not uniform and can vary significantly depending on several sociodemographic factors, including age, gender, residence, and socioeconomic status (14). These factors can either exacerbate or mitigate the impact of functional impairments on mental health, creating a complex and multifaceted association. For example, studies indicate that women often experience a stronger association between functional limitations and depressive symptoms compared to men, potentially due to differences in social roles, caregiving responsibilities, and coping mechanisms (15). Women, particularly those who have traditionally served as caregivers, may experience a more pronounced sense of loss when their independence is compromised, leading to heightened psychological distress and vulnerability to depressive symptoms. Similarly, older adults with lower socioeconomic status face greater psychological challenges related to functional limitations, as restricted financial resources often limit their access to healthcare, social support services, and resources that could alleviate their physical and mental health burdens (16).

In China, urban-rural disparities introduce an additional layer of complexity to this relationship (17, 18). Older adults in rural areas frequently encounter barriers to accessing healthcare and social services, which can amplify the mental health impacts of physical impairments, while their urban counterparts may benefit from better healthcare infrastructure and more available support networks (19). Rural older adults may rely heavily on family support due to limited public health resources, which places an additional burden on families and may increase the psychological stress associated with physical limitations (20). In contrast, urban residents may have greater access to medical facilities, mental health professionals, and social programs designed to support older adults, allowing for more effective management of both functional limitations and mental health needs (20). Thus, the urban-rural divide in healthcare access plays a critical role in shaping the experience of functional impairments and their mental health implications in the aging population (21).

The Ecological Model of Health Behavior, as originally proposed by Bronfenbrenner (22), delineates five hierarchical levels of influence on health: individual, interpersonal, organizational, community, and policy (societal). However, in empirical research and applied intervention studies, this model is frequently conceptually condensed into three primary levels to facilitate analytical clarity and practical application. These commonly adopted tiers include: the individual level (e.g., biological and personal factors), the interpersonal or familial level (e.g., social relationships and household dynamics), and the societal or structural level (e.g., institutional resources, community context, and policy environment). This tripartite framework is especially useful in public health research for identifying multilevel intervention targets and understanding how health outcomes are shaped by nested environmental systems.

This study adopts a tripartite ecological framework grounded in Bronfenbrenner’s ecological systems theory (1979), and further informed by McLeroy et al.’s (23) ecological model of health promotion. While the original ecological theory includes multiple nested systems, contemporary health behavior research often categorizes them into three analytically tractable layers—individual, familial, and societal (24, 25). These levels allow for a nuanced analysis of how personal characteristics (e.g., age), family context (e.g., living arrangement), and social-structural environment (e.g., urban-rural region) interact to influence mental health outcomes among older adults. Building on this theoretical framework, the present study examines how specific sociodemographic characteristics—categorized across individual, familial, and societal domains—moderate the relationship between functional limitations and depressive symptoms in later life.

The role of sociodemographic factors such as age, residence, and living arrangements in the relationship between functional limitations and depressive symptoms highlights the need for a nuanced understanding of how these factors intersect with health outcomes in the aging population (26, 27). Age itself is a critical factor, as functional limitations and the severity of depressive symptoms tend to increase with age, compounding the impact on overall well-being, with older adults often experiencing multiple chronic conditions that exacerbate functional limitations and contribute to higher levels of depressive symptoms (3, 16). Additionally, social isolation frequently accompanies aging, further increasing the risk of depressive symptoms, particularly in rural areas where limited social support and healthcare resources are common (28). Urban-rural differences also shape this association, as urban older adults generally have better access to healthcare and social services, whereas rural older adults often experience restricted access, intensifying the psychological effects of functional impairments (29). Living arrangements—whether living alone, with a spouse, or with extended family—impact both social and instrumental support levels, which can mediate the extent to which functional limitations contribute to depressive symptoms (30, 31). Socioeconomic status plays an equally crucial role, as higher income levels generally enable broader access to health services, assistive devices, and social engagement, all of which help mitigate the psychological impact of functional limitations (20).

Understanding the intricate relationship between functional limitations and depressive symptoms, as well as the moderating effects of sociodemographic factors, is essential for developing effective support systems and targeted interventions (32). By recognizing the impact of functional limitations on mental health outcomes, healthcare providers and policymakers can create interventions tailored to the unique needs of older adults facing these impairments, which can be particularly effective given the increasing prevalence of functional limitations in aging populations (33). For instance, enhancing access to assistive technologies, mental health support services, and programs encouraging social engagement has been shown to reduce the psychological burden of functional limitations and significantly improve quality of life among older adults. Additionally, addressing underlying sociodemographic inequalities in healthcare access and support systems is crucial to ensuring that interventions reach the most vulnerable groups, including those in rural or low-income areas, who often experience compounded challenges related to both physical impairments and limited social resources (34).

The extensive evidence linking functional limitations and depressive symptoms among older adults, while well-documented, highlights the need for further exploration of sociodemographic moderators that could refine our understanding of this connection (35, 36, 59). Variables such as gender, age, education, and socioeconomic status have shown potential as moderators of the effects of functional limitations on depressive symptoms. Understanding how these factors interact with functional limitations to influence depressive symptoms could offer valuable insights into the mental health disparities observed among older adults (37). For example, educational attainment might equip individuals with better coping strategies and greater awareness of available resources, potentially mitigating some of the depressive symptoms associated with functional limitations. Similarly, individuals with higher socioeconomic status may experience fewer psychological burdens associated with functional impairments due to enhanced access to healthcare services, assistive devices, and social support networks. This study investigates the moderating effects of sociodemographic factors selected through a tripartite ecological framework encompassing individual (age group), familial (living arrangement), and societal (urban-rural region) levels on the association between functional limitations and depressive symptoms among Chinese older adults (22).

This study aims to examine the moderating effects of sociodemographic factors on the relationship between functional limitations and depressive symptoms among older Chinese adults. By investigating these interactions, we seek to identify specific subgroups that may be at greater risk of experiencing depressive symptoms in the presence of functional limitations. The findings from this research are expected to deepen our understanding of the complex interactions between physical health, sociodemographic characteristics, and mental well-being in aging populations. This knowledge will be instrumental in developing culturally and contextually appropriate interventions and healthcare policies, ultimately improving mental health outcomes for vulnerable subgroups within the older adult population.

By addressing the role of sociodemographic factors, this study contributes to a more comprehensive understanding of the multifaceted influences on depressive symptoms among older adults facing functional limitations. Through insights gained from this research, healthcare providers, policymakers, and caregivers will be better equipped to design targeted interventions that recognize and address the unique needs of this population. Such interventions are critical in enhancing the quality of life, promoting mental well-being, and ensuring equitable access to care and support for older adults, particularly in the context of an aging society where functional limitations and mental health issues are increasingly prevalent.

## 2 Methods

### 2.1 Data and study population

We utilized data from the Chinese Longitudinal Healthy Longevity Survey (CLHLS), a large-scale, ongoing prospective cohort study that covers 23 of China’s 31 provinces. The CLHLS was established in 1998 to investigate factors influencing healthy aging and longevity among the Chinese population, making it one of the most comprehensive longitudinal studies on aging in China. This cohort study has had subsequent follow-ups and participant recruitment waves in 2000, 2002, 2005, 2008, 2011, 2014, and 2018. Detailed information on the study design, sampling, and methodology has been documented extensively in prior publications (38).

For the present analysis, we included data from the most recent follow-up wave in 2018, which allowed us to examine the transitions in health and well-being over this four-year period. The CLHLS collects a wide array of data, including sociodemographic characteristics, lifestyle factors, cognitive and physical health assessments, and functional and psychological well-being, thus providing a robust foundation for analyzing factors that influence aging outcomes in older Chinese adults.

This study received approval from the Research Ethics Committee of Peking University (IRB00001052-13074), and all participants or their legal representatives provided informed consent through a signed form, confirming their willingness to participate in the baseline and follow-up surveys.

After excluding participants with missing data, a final sample of 9,354 older adults aged 65 years and above was included in this analysis. This large sample enhances the statistical power of our study and allows for a more precise estimation of associations related to aging and health outcomes in the Chinese population.

### 2.2 Measurements

#### 2.2.1 Dependent variable

The dependent variable was depressive symptoms measured using the 10-item Center for Epidemiologic Studies Depression Scale (CES-D-10) (39). This scale is a widely used survey tool to measure depressive symptoms among Chinese older adults, with good validity and reliability. All items are rated on a four-point scale, from “rarely” to “on some days” (1–2 days), “occasionally” (3–4 days), or “most of the time” (5–7 days). We scored the responses of “rarely,” “on some days” (1–2 days), “occasionally” (3–4 days), and “most of the time” (5–7 days) as 0, 1, 2, and 3, respectively, after we reversely coded the responses to two positive questions— “I was happy” and “I felt hopeful about the future.” The total range of CES-D-10 scores is 0–30, with higher scores indicating a greater severity of depressive symptoms. The well-validated cutoff value is 10 in measuring depressive symptoms among Chinese older populations (40, 41); therefore, participants with a score ≥ 10 on the CES-D-10 were considered to have depressive symptoms.

#### 2.2.2 Independent variable

In this study, the independent variable is functional limitations, which refer to reductions in the capacity to engage in activities of daily living (ADLs) or instrumental activities of daily living (IADLs) due to physical, cognitive, or emotional challenges. Within the CLHLS questionnaire, participants’ ADLs were evaluated based on their reported difficulty in performing tasks such as dressing, bathing/showering, eating, getting into or out of bed, using the toilet, and controlling urination and defecation. For each of these six tasks, participants were provided with the following response options: (1) no, I do not have any difficulty; (2) I have difficulty but can still manage; (3) yes, I have difficulty and require assistance; (4) I am unable to perform the task. Consistent with the approach employed by Zhang et al. (60), We scored the responses of “(1) no, I do not have any difficulty,” “(2) I have difficulty but can still manage”, “(3) yes, I have difficulty and require assistance”, and “(4) I am unable to perform the task” as 0, 1, 2, and 3. By aggregating responses to these six tasks, we obtained ADL limitations ranging from 0-18. Regarding IADLs, participants were queried about their difficulty in preparing hot meals, shopping for groceries, making phone calls, taking medication, and managing money. Following the same operationalization as ADLs, we obtained IADL limitations ranging from 0-15. We collectively refer to ADL and IADL limitations as functional limitations, with a range from 0 to 33.

#### 2.2.3 Moderating variables

This study examines three key sociodemographic moderating factors—age group, living arrangement, and region—that reflect individual, familial, and society relevant to aging outcomes. By categorizing age into “young-old” (65–74), “old-old” (75-84) and “oldest old” (85+), differentiating living arrangements between those living alone versus with family, and distinguishing between urban and rural regions, the study seeks to reveal how these contextual variables shape health and well-being among older adults.

#### 2.2.4 Control variables

Control variables in this study included a range of demographic factors such as age, gender, marital status, household income, and perceived social stratification. Age, gender, and marital status provide insights into basic sociodemographic profiles that can influence health outcomes and psychological well-being. Household income, a crucial socioeconomic indicator, reflects the material resources available to individuals and can impact access to healthcare, social services, and general quality of life among older adults. Perceived social stratification was assessed through a single-item measure that captures an individual’s self-reported economic standing relative to others in their neighborhood. Respondents were asked, “How would you rate your economic status compared to other people in your neighborhood?” with responses ranging from 1 (“very rich”) to 5 (“very poor”). This self-assessment allows for the examination of subjective socioeconomic status, which has been shown to be a meaningful predictor of health outcomes, as it often reflects perceived social inequality and related stressors (42, 43). Including these control variables helps to account for underlying demographic and socioeconomic influences that may affect the primary variables under study, thus improving the robustness of the analysis.

### 2.3 Data analysis

**Figure 1:**
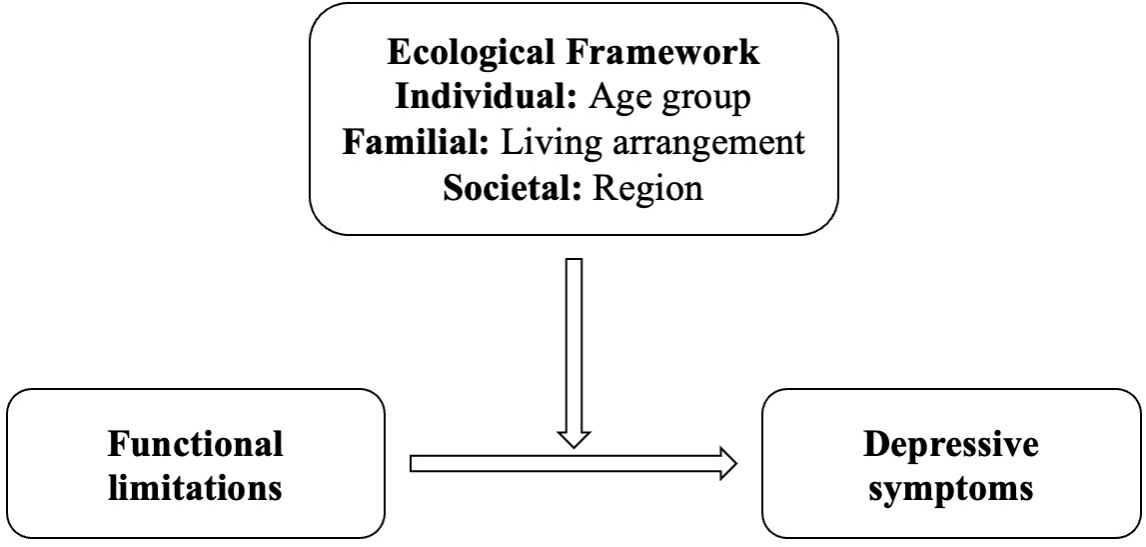
Research model.

The present study was primarily carried out to investigate the effect of functional limitations on depressive symptoms among older Chinese adults, and the potential moderating role of sociodemographic factors were also explored (**Figure 1**). To accomplish this, statistical analysis was conducted using STATA 17 software. Specifically, a three-step analytical approach was employed. Firstly, descriptive analysis was performed to illustrate the general characteristics of the study population, generating a summary of the relevant demographic and clinical variables, such as age, gender, region, and marital status. Secondly, correlation analysis was utilized to examine the relationships between the key study variables, namely functional limitations, depressive symptoms, and sociodemographic factors, so as to assess the strength and direction of these relationships, and to identify any potential confounding factors that might affect the main study outcomes. Finally, regression analysis was employed to investigate the impact of interaction terms (i.e. moderating effects) on depressive symptoms, while controlling for potential confounding factors identified during the correlation analysis stage, for which, a series of regression models were constructed to examine the main and interactive effects of functional limitations, sociodemographic factors, and other relevant covariates on depressive symptoms. Overall, this analytical approach allowed for a thorough examination of the research questions and provided valuable insights into the role of functional limitations and sociodemographic factors in depressive symptoms among older Chinese adults.

## 3 Results

### 3.1 Characteristics of the study population

The sample characteristics in **Table 1** reveal significant sociodemographic, health behavior, and health status variations among 9,354 older Chinese adults. The majority are female (53.86%) and “oldest-old” (43.39%), with women more likely to live alone, particularly in rural areas where functional limitations and depressive symptoms are more prevalent. Education levels differ substantially between age groups and regions; only 44.07% are unschooled, with a greater proportion of educated individuals in the younger elderly group and urban areas. Most participants are nonsmokers (83.80%) and nondrinkers (84.50%), with rural residents and younger elderly individuals more likely to report smoking and drinking. Regular exercise is reported by 34.92%, with higher rates among urban residents and the younger elderly. Perceived economic status, a key subjective socioeconomic indicator, is higher among urban residents and those living with family, while those living alone and rural participants report slightly lower perceived economic status. Functional limitations average 5.18, with higher levels among the old elderly and rural residents. Depression scores are similarly elevated in rural areas and among those living alone, emphasizing the health vulnerabilities within specific subgroups. These patterns underscore the importance of targeted health and support interventions for older Chinese adults, particularly those in rural settings or experiencing social isolation.

**Table 1:** Sample characteristics.

Variables	N	Freq (%) /Mean (SD)	Age group			Living arrangement		Region	
			Young-old	Old-old	Oldest-old	Living alone	Living with household member	Urban	Rural
Gender									
Male	4,316	46.14	1,289 (50.33)	1,337 (48.90)	1,690 (41.64)	600 (37.76)	3,716 (47.86)	1,366 (49.62)	2,950 (44.69)
Female	5,038	53.86	1,272 (49.67)	1,397 (51.10)	2,369 (58.36)	989 (62.24)	4,049 (52.14)	1,387 (50.38)	3,651 (55.31)
Education									
Unschooled	4,122	44.07	538 (21.01)	1,067 (39.03)	2,517 (62.01)	832 (52.36)	3,290 (42.37)	648 (23.54)	3,474 (52.63)
Schooled	5,232	55.93	2,023 (78.99)	1,667 (60.97)	1,524 (37.99)	757 (47.64)	4,475 (57.63)	2,105 (76.46)	3,127 (47.37)
Smoking									
No	7,839	83.80	1,992 (77.78)	2,275 (83.21)	3,572 (88.00)	1,355 (85.27)	6,484 (83.50)	2,419 (87.87)	5,420 (82.11)
Yes	1,515	16.20	569 (22.22)	459 (16.79)	487 (12.00)	234 (14.73)	1,281 (16.50)	334 (12.13)	1,181 (17.89)
Drinking									
No	7,904	84.50	2,034 (79.42)	2,290 (83.76)	3,580 (88.20)	1,356 (85.34)	6,548 (84.33)	2,368 (86.02)	5,536 (83.87)
Yes	1,450	15.50	527 (20.58)	444 (16.24)	479 (11.80)	233 (14.66)	1,217 (15.67)	385 (13.98)	1,065 (16.13)
Exercise									
No	6,088	65.08	1,436 (56.07)	1,599 (58.49)	3,053 (75.22)	1,051 (66.14)	5,037 (64.87)	1,376 (49.98)	4,712 (71.38)
Yes	3,266	34.92	1,125 (43.93)	1,135 (41.51)	1,006 (24.78)	538 (33.86)	2,728 (35.13)	1,377 (50.02)	1,889 (28.62)
Perceived economic status	9,354	3.12 (.64)	3.10 (.61)	3.11 (.65)	3.14 (.65)	3.03 (.65)	3.14 (.63)	3.31 (.65)	3.04 (.61)
Age group									
Young-old	2,561	27.38				268 (16.87)	2,293 (29.53)	746 (27.10)	1,815 (27.50)
Old-old	2,734	29.23				561 (35.31)	2,173 (27.98)	800 (29.06)	1,934 (29.30)
Oldest-old	4,059	43.39				760 (47.83)	3,299 (42.49)	1,207 (43.84)	2,852 (43.21)
Living arrangement									
Living alone	1,589	16.99	268 (10.46)	561 (20.52)	760 (18.72)			401 (14.57)	1,188 (18.00)
With household member	7,765	83.01	2,293 (89.54)	2,173 (79.48)	3,299 (81.28)			2,352 (85.43)	5,413 (82.00)
Region									
Urban	2,753	29.43	746 (29.13)	800 (29.26)	1,207 (29.74)	401 (25.24)	2,352 (30.29)		
Rural	6,601	70.57	1,815 (70.87)	1,934 (70.74)	2,852 (70.26)	1,188 (74.76)	5,413 (69.71)		
Functional limitations	9,354	5.18 (6.88)	.86 (2.65)	2.76 (4.50)	9.53 (7.51)	3.99 (5.25)	5.42 (7.14)	5.50 (7.41)	5.04 (6.64)
Depressive symptoms	9,354	12.13 (6.11)	11.18 (6.04)	12.11 (6.06)	12.75 (6.11)	13.44 (6.37)	11.87 (6.02)	11.24 (6.27)	12.51 (6.00)

### 3.2 Correlation of the main variables

**Table 2** presents the correlations between the main variables, including depressive symptoms, age group, living arrangement, region, and functional limitations. depressive symptoms show a positive correlation with both age group (r = 1.104, p <.001) and region (r = .095, p <.001), suggesting that depressive symptoms are slightly higher among the “oldest- old” (85+) and rural residents. depressive symptoms also demonstrate a moderate positive correlation with functional limitations (r = .211, p <.001), indicating that greater physical limitations are associated with higher depressive symptoms. Conversely, a negative correlation is observed between depressive symptoms and living arrangement (r = -.097, p <.001), implying that individuals living alone tend to report higher levels of depressive symptoms.

**Table 2:** Correlation of the main variables.

Variables	(1)	(2)	(3)	(4)	(5)	Mean	SD	Range
(1) Depressive symptoms	1.000					12.13	6.11	0-30
(2) Age group	.104***	1.000				1.16	.83	0-2
(3) Living arrangement	-.097***	-.082***	1.000			.83	.38	0-1
(4) Region	.095***	-.006	-.042**	1.000		.71	.46	0-1
(5) Functional limitations	.211***	.540***	.078***	-.030**	1.000	5.18	6.88	0-28

**p<0.01; ***p<0.001.

Functional limitations show the strongest correlation with age group (r = .540, p <.001), highlighting that physical limitations are more common among older participants. Functional limitations are also positively correlated with living arrangement (r = .078, p <.001) and negatively with region (r = -.030, p <.01), suggesting that limitations are more pronounced among those living alone and slightly lower among rural residents. Additionally, age group and living arrangement exhibit a negative correlation (r = -.082, p <.001), indicating that older participants are more likely to live with family members. Overall, the correlations suggest that age, living arrangement, region, and functional limitations are interrelated and may collectively impact depression levels among older adults.

### 3.3 Moderation model

**Table 3** presents the moderation analysis of sociodemographic factors (age group, living arrangement, and region) on the relationship between functional limitations and depressive symptoms. The models progressively incorporate these moderators and their interaction effects, allowing for a nuanced examination of how sociodemographic factors influence the association between functional limitations and depressive symptoms. The F-values in **Table 3** indicate the overall fit and statistical significance of each model in explaining the relationship between functional limitations and depressive symptoms, with sociodemographic moderators included. Each model displays a statistically significant F-value (p < 0.001), suggesting that the variables collectively explain a meaningful portion of the variance in depressive symptoms among older adults.

**Table 3:** Moderation analysis of sociodemographic factors on the relationship between functional limitations and depressive symptoms.

Variables	Model 1		Model 2		Model 3		Model 4	
	β	SE	β	SE	β	SE	β	SE
Constant	29.559***	.395	28.545***	.398	28.650***	.542	30.621***	.497
Gender	.344**	.139	.344**	.138	.297**	.138	.308**	.138
Education	-.869***	.134	-.469***	.136	-.279*	.143	-.250*	.143
Smoking	-.351**	.178	-.168	.176	-.193	.176	-.149	.176
Drinking	-1.145***	.176	-.987***	.175	-.999***	.175	-.977***	.174
Exercise	-1.870***	.129	-1.431***	.132	-1.351***	.134	-1.406***	.134
Perceived economic status	-2.109***	.095	-2.134***	.094	-2.049***	.095	-2.023***	.095
Functional limitations			.127***	.009	.139***	.010	.361***	.052
Age group					-.047	.149	-.816***	.230
Living arrangement					-1.419***	.160	-1.369***	.163
Region					.386***	.140	.349**	.140
Functional limitations×Age group							-.127***	.017
Functional limitations×Living arrangement							-.058*	.029
Functional limitations×Region							.059***	.018
R^2^	.106		.124		.132		.139	
Adj R^2^	.106		.123		.131		.138	
F	185.2***		188.4***		141.9***		116.20***	

*p<0.05; **p<0.01; ***p<0.001.

In Model 1, baseline demographic variables (gender, education, smoking, drinking, exercise, and perceived economic status) are included. Gender and perceived economic status show significant effects on depressive symptoms, with women and those perceiving a lower economic status reporting higher depression scores. Education, exercise, and drinking are negatively associated with depressive symptoms, suggesting that higher educational attainment, physical activity, and alcohol consumption are linked to lower levels of depressive symptoms. This model explains 10.6% of the variance in depression scores (R² = .106, p <.001).

In Model 2, functional limitations are added, demonstrating a significant positive association (β = .127, SE = .009, p <.001), indicating that higher levels of functional limitations are associated with increased depressive symptoms. This model slightly improves the explained variance (R² = .124).

Model 3 incorporates the sociodemographic factors age group, living arrangement, and region. The results reveal significant effects for living arrangement (β = -1.419, SE = .160, p <.001) and region (β = .386, SE = .140, p <.01). Older adults living alone report higher depression scores, while those in rural regions also show increased depressive symptoms. The inclusion of these moderators further increases the model’s explanatory power (R² = .132).

Finally, Model 4 includes interaction terms between functional limitations and each sociodemographic factor to test for moderation effects. The results confirm significant interaction effects between functional limitations and age group (β = -.127, SE = .017, p <.001), living arrangement (β = -.058, SE = .029, p <.01), and region (β = .059, SE = .018, p <.001). These interactions indicate that the impact of functional limitations on depressive symptoms varies by sociodemographic context: the association is stronger in younger elderly, those living alone, and individuals in rural regions. Model 4 explains the highest variance in depression scores, with an R² of.139.

**Figure 2:**
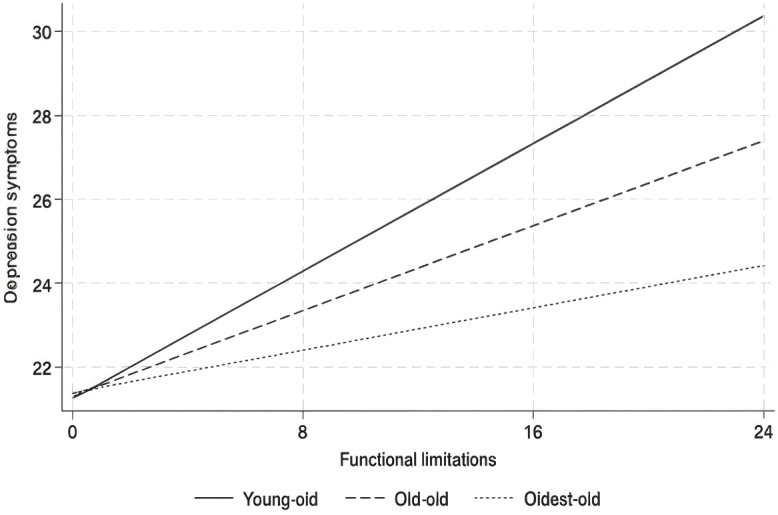
Moderation analysis of the interaction between functional limitations and age group on depressive symptoms.

**Figure 3:**
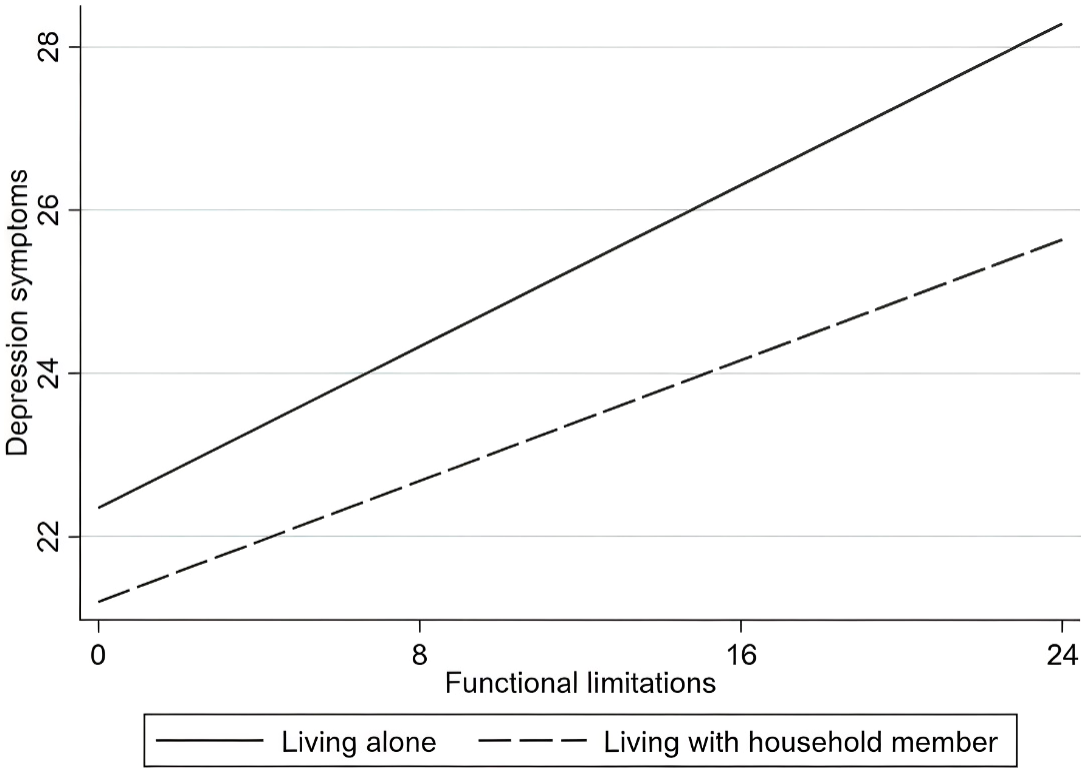
Moderation analysis of the interaction between functional limitations and living arrangement on depressive symptoms.

**Figure 4:**
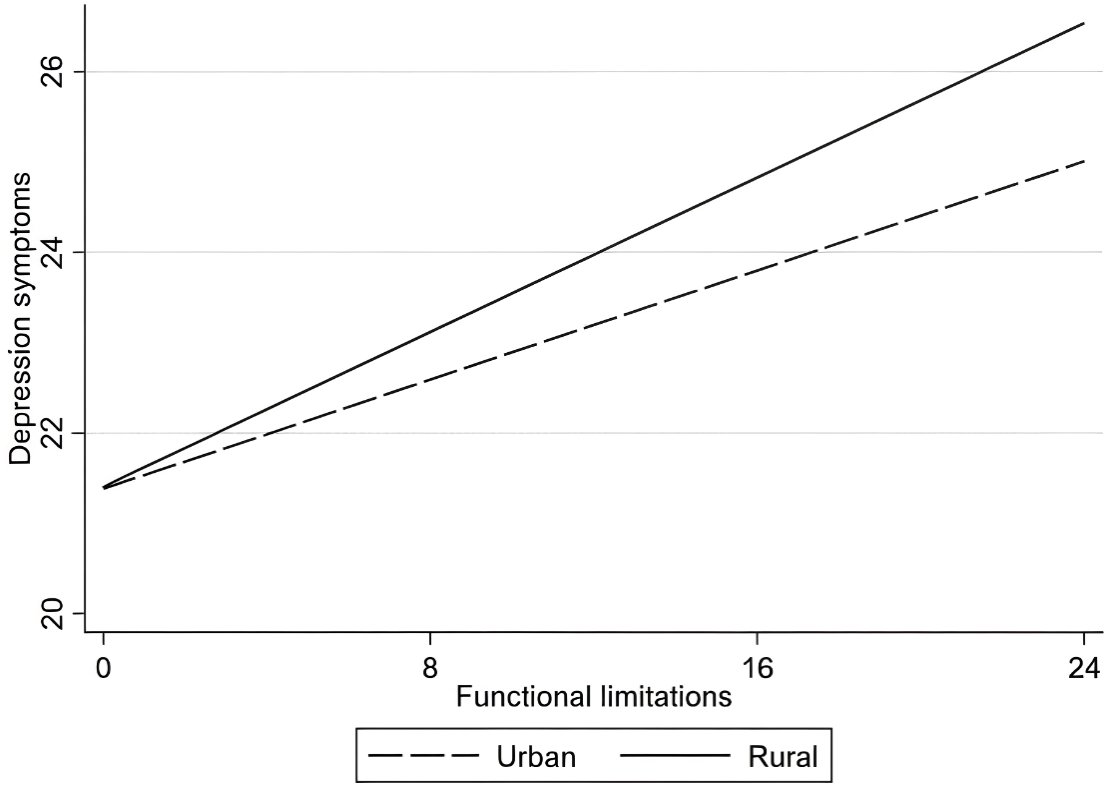
Moderation analysis of the interaction between functional limitations and region on depressive symptoms.

The specific moderating effects are as follows. First, within the age group variable, functional limitations have a greater impact on depressive symptoms among the “young-old” compared to the “oldest-old,” as shown in **Figure 2**. Second, for living arrangements, the effect of functional limitations on depressive symptoms is more pronounced among older adults living alone than among those residing with family members, as illustrated in **Figure 3**. Finally, in terms of region, functional limitations have a stronger effect on depressive symptoms among rural older adults than their urban counterparts, as depicted in **Figure 4**. Overall, the findings suggest that sociodemographic factors such as age group, living arrangement, and region significantly moderate the relationship between functional limitations and depressive symptoms among older Chinese adults.

## 4 Discussion

This study investigates how sociodemographic factors—specifically age group, living arrangement, and region—moderate the relationship between functional limitations and depressive symptoms among older Chinese adults. The findings reveal nuanced interactions between these variables, demonstrating that sociodemographic contexts significantly shape how functional limitations influence depressive symptoms. Below, we discuss the implications of each moderating variable in the context of existing literature, the potential mechanisms driving these effects, and the broader implications for interventions and policy.

The results indicate that the impact of functional limitations on depressive symptoms is more pronounced among the “young-old” (65–74) compared to the “old-old” (75-84) and “oldest-old” (85+), aligning with evidence that younger older adults may experience greater psychological distress from functional decline due to a disruption of previously active lifestyles (44, 45). This contrast suggests that while the young-old often view these limitations as unexpected, the old-old and oldest-old may have adjusted to age-related changes, seeing physical decline as a natural part of aging and, therefore, experiencing less associated psychological distress (46, 47). This age-related moderation underscores the need for tailored interventions: younger older adults could benefit from initiatives focusing on preserving autonomy through physical rehabilitation, mobility aids, and assistive technologies designed to maintain independence (48). For the old-old, support should emphasize social and psychological resilience, helping them cope with age-related limitations while fostering emotional well-being through social support networks (49). Addressing these age-specific needs may optimize mental health outcomes for both age groups by recognizing and catering to their distinct perceptions and experiences of functional limitations.

The findings further reveal that functional limitations have a stronger impact on depressive symptoms among those living alone compared to those living with family (50). This result is consistent with prior research emphasizing the buffering effect of social support against depressive symptoms (51). Family members often provide practical assistance with daily tasks, emotional support, and companionship, all of which can mitigate the depressive impact of functional limitations (52). For older adults living alone, however, functional limitations may exacerbate feelings of loneliness, helplessness, and isolation, which are closely linked to depressive symptoms (53). This finding underscores the importance of social support systems and suggests that policies should focus on enhancing community and social services for older adults who live alone. Programs aimed at improving social interaction, such as volunteer and mentorship programs, could help reduce isolation and provide emotional support. Additionally, strengthening in-home care services and ensuring access to community centers for older adults living alone could alleviate the adverse effects of functional limitations on mental health.

Finally, the study highlights that functional limitations have a more substantial effect on depressive symptoms among rural older adults than urban ones. This finding can be attributed to disparities in healthcare infrastructure, accessibility to mental health resources, and social services between urban and rural areas in China (54). Rural residents may have fewer resources to manage physical limitations, limited access to mental health services, and reduced availability of community-based support, which together exacerbate the psychological toll of functional limitations. Addressing this regional disparity requires a focus on policy measures that enhance healthcare access and social support in rural areas. Policymakers should consider prioritizing rural healthcare funding, particularly for programs that target older adults with functional limitations. Community-based support networks and mobile healthcare units could bridge gaps in service delivery, bringing essential healthcare resources directly to rural communities. Additionally, telemedicine and mental health counseling services could provide accessible and affordable support for older adults in these areas, helping to mitigate the depressive effects associated with functional limitations.

The findings of this study have implications for theories that address the intersection of physical health and mental well-being in aging populations. The Life Course Theory, which emphasizes the influence of early life conditions and accumulated experiences on later-life health outcomes, may help explain why younger older adults exhibit greater distress in response to functional limitations than their older counterparts (55). For the young-old, functional limitations may signify a break from their previously active roles and aspirations, which can be psychologically unsettling. Resilience Theory further supports the idea that older adults living alone or in rural areas may lack the social and structural resources necessary to cope with the stress associated with functional decline, thereby heightening their risk of depressive symptoms (56, 57). These findings contribute to a nuanced understanding of how sociodemographic factors impact the association between physical limitations and depressive symptoms, suggesting that the relationship is not only a matter of individual health but is significantly shaped by social, familial, and environmental factors. Integrating these theories can provide a comprehensive framework for understanding the complex interactions between functional limitations and depressive symptoms in aging populations, particularly in culturally specific contexts like China.

From a practical perspective, this study highlights the need for targeted mental health interventions and social policies that address the sociodemographic diversity within the older adult population. First, healthcare practitioners working with Chinese older adults should consider the sociodemographic context when assessing and treating depressive symptoms in individuals with functional limitations. Tailored interventions that address specific needs based on age, living arrangement, and region can improve mental health outcomes and promote overall well-being (58, 60). For younger older adults with functional limitations, practitioners could focus on empowering strategies that maintain their sense of autonomy and prevent a decline in psychological well-being. For older adults living alone, building robust social support networks through community initiatives could help alleviate feelings of loneliness and reduce the risk of depressive symptoms. Lastly, for rural residents, expanding access to healthcare resources and strengthening local support services are critical steps toward reducing health disparities and improving mental health among older adults. Policymakers should prioritize healthcare accessibility in rural areas, increase funding for mental health services, and foster community-based programs that support independent living for older adults. Additionally, policies that promote age-friendly environments—such as accessible transportation, safe housing, and community centers—can play an essential role in fostering healthy aging and reducing the negative mental health impacts associated with functional limitations.

While this study provides valuable insights into the moderating effects of sociodemographic factors, there are some limitations. First, the study’s reliance on self-reported measures may introduce bias, as older adults may underreport depressive symptoms or functional limitations due to social desirability or memory constraints. Future research could incorporate more objective measures, such as clinical assessments, to obtain a more accurate understanding of the impact of functional limitations on mental health. Additionally, this study focuses on cross-sectional data, limiting the ability to draw causal conclusions about the relationship between functional limitations and depressive symptoms. Longitudinal research could provide further insight into how this relationship evolves over time and how sociodemographic factors may impact this trajectory. Furthermore, given the focus on Chinese older adults, it would be beneficial to replicate this study in other cultural settings to assess the generalizability of these findings. While this study focused on three multilevel moderators, future research could examine additional factors (e.g., gender, education) using advanced modeling techniques. Nevertheless, our findings provide actionable insights for policymakers to design stratified interventions across individual, familial, and societal dimensions.

In conclusion, this study sheds light on the complex interplay between functional limitations and depressive symptoms among Chinese older adults, with sociodemographic factors playing a significant moderating role. Age group, living arrangement, and region each influence the strength of the relationship between functional limitations and depressive symptoms, underscoring the need for targeted interventions that consider the individual, familial, and society of aging. By focusing on the unique needs of subgroups within the older adult population, healthcare providers and policymakers can better support mental health and enhance quality of life in this vulnerable population.

## 5 Conclusion

This study investigated the role of sociodemographic moderators—age group, living arrangement, and region—in the relationship between functional limitations and depressive symptoms among Chinese older adults. The findings underscore that functional limitations have a differentiated impact on depressive symptoms depending on these key sociodemographic factors. Specifically, functional limitations are more strongly associated with depressive symptoms in the “young-old” group than in the “oldest-old,” suggesting that younger older adults may experience a sharper decline in mental well-being as physical impairments arise. Additionally, older adults living alone show a greater vulnerability to the depressive effects of functional limitations compared to those residing with family members, likely due to reduced social support. Regionally, rural older adults exhibit a stronger association between functional limitations and depressive symptoms than urban residents, highlighting the role of limited healthcare access and support services in rural areas.

Overall, the results indicate that sociodemographic factors significantly shape the psychological impact of functional limitations. These insights emphasize the need for targeted interventions that consider age, living arrangement, and regional differences, which can improve mental health outcomes by addressing the specific needs of diverse subgroups within China’s aging population.

## Data Availability

Publicly available datasets were analyzed in this study. This data can be found here: China Longitudinal Healthy Longevity Survey, https://opendata.pku.edu.cn/dataverse/CHADS.
